# A novel mutation in *FNIP1* associated with a syndromic immunodeficiency and cardiomyopathy

**DOI:** 10.1007/s00251-024-01359-3

**Published:** 2024-11-14

**Authors:** Ilia Spivak, Atar Lev, Amos J. Simon, Ortal Barel, Ido Somekh, Raz Somech

**Affiliations:** 1 Clinical Immunology, Angioedema and Allergy Institute, The Center for Autoimmune Diseases, Sheba Medical Center, Tel-Hashomer, Israel; 2 Cancer Research Center, Pediatric Immunology Unit, Jeffrey Modell Foundation (JMF) Center, Department of Pediatrics, Edmond and Lily Safra Children’s Hospital, Sheba Medical Center, 52621 Tel Hashomer, Israel; 3 The Genomic Unit, Sheba Cancer Research Center, Sheba Medical Center, Tel Hashomer, Israel; 4 Faculty of Medicine, Tel Aviv University, Tel Aviv, Israel; 5 Pediatric Department A and Immunology Service, Jeffrey Modell Foundation Center, Edmond and Lily Safra Children’s Hospital, Sheba Medical Center, Tel Hashomer, Israel

**Keywords:** Primary immunodeficiency, *FNIP1*, Cardiomyopathy

## Abstract

Genetic variants in *Folliculin interacting protein 1 (FNIP1)* were recently discovered as monogenic causes for immunodeficiency and cardiomyopathy, with only a few patients diagnosed thus far. In this study, we describe a patient harboring a novel genetic variant in *FNIP1* causing immunodeficiency with cardiac involvement. Clinical and immunological workups were performed. Genetic evaluation utilizing whole-exome sequencing (WES) and Sanger sequencing was conducted. The index patient (subject II-4) presented with hypertrophic cardiomyopathy, recurrent infections, and chronic diarrhea during infancy. Immune workup revealed agammaglobulinemia and a lack of B lymphocytes. Genetic evaluation identified a homozygous 13-bp duplication variant in *FNIP1* (c.52_64dupGCGCCCGGCCGCG, p. Asp22GlyfsTer21) resulting in a frameshift in exon 1/18. She was treated with supplemental intravenous immunoglobulins (IVIg) with good control of sinopulmonary and gastrointestinal manifestations. Her sibling (subject II-1) had similar clinical features, along with dysmorphic facial features and hypotony, and succumbed to cardiogenic shock at the age of 2 months, prior to genetic evaluation. Diagnosis of novel immunodeficiencies promotes our understanding of the immune system, enabling genetic counseling as herein, and may assist in the development of novel medical therapies in the future. FNIP1 loss-of-function should be considered in patients presenting in infancy with cardiac manifestations along with agammaglobulinemia (and B-cell lymphopenia).

## Introduction

Inborn errors of immunity (IEI) comprise a heterogeneous group of disorders of the immune system predisposing affected individuals to acquire recurrent and/or severe infections, allergy, malignancy, autoinflammation, and autoimmune manifestations (Bucciol et al. 2024; Fischer et al. 2017; Notarangelo et al. 2020). Currently, 485 genetic causes of IEI have been included by the International Union of Immunological Societies (IUIS) expert committee. These diseases encompass disorders of B-cells and/or T-cells, the innate system, NK-cells, neutrophil defects, complement disorders, immune dysregulation disorders, auto-inflammatory diseases, bone marrow failure syndromes, and syndromic IEI (McCusker et al. 2018; Tangye et al. 2022; Yu 2024).

FNIP1 loss-of-function (LOF) is a monogenic disease-causing disorder that results in hypogammaglobulinemia with syndromic features. *Folliculin interacting protein 1* (*FNIP1*) encodes the FNIP1 protein, a regulator of adenosine monophosphate-activated protein (AMPK) and mammalian target of rapamycin (mTOR) cellular pathways (Deenick et al. 2020). FNIP1 regulates mitochondrial activity and is crucial for B-cell maturation and myocardial function in animal models (Niehues et al. 2020; Saettini et al. 2021). Animal studies conducted on neonatal hearts from knockout *FNIP1* -/- mice suggest that FNIP1 may inhibit AMPK function and cause mTOR activation. These cellular processes in cardiomyocytes lead to the accumulation of glycogen, causing cardiomyopathy and conduction disorders (Siggs et al. 2016). FNIP1 LOF in humans is associated with immunodeficiency characterized by hypogammaglobulinemia, neutropenia, and recurrent infections along with cardiac involvement, including hypertrophic cardiomyopathy (HCM), tachyarrhythmias, and pre-excitation syndromes (Niehues et al. 2020; Saettini et al. 2021). Additional features reported in patients with FNIP1 LOF include myopathy, renal cysts (Deenick et al. 2020), and central nervous involvement (developmental delay, microcephaly, and others) (Moreno-Corona et al. 2023; Niehues et al. 2020). To date, seven patients with *FNIP1*-associated immunodeficiency have been reported in the literature, most of whom presented typical clinical features during infancy (Deenick et al. 2020; Moreno-Corona et al. 2023; Niehues et al. 2020; Park et al. 2008; Saettini et al. 2021; Yazdani et al. 2020). In this study, we aim to describe a novel genetic variant in *FNIP1*, causing immunodeficiency with cardiac involvement.

## Methods

### Patients and clinical evaluation

Clinical data were collected and reviewed from digital hospital-based data. All the procedures were performed following informed consent from the patient parents, in accordance with the ethical standards of the institutional and/or national research committees and with the current update of the Declaration of Helsinki.

### Immunological evaluation

#### Lymphocyte markers

Cell surface marker expression of peripheral blood mononuclear cells (PBMC) was analyzed by immunofluorescent staining with monoclonal antibodies and flow cytometry (Epics V; Coulter Electronics, Hialeah, FL) (Lev et al. 2012; Somekh et al. 2019, 2021).

### Genetic evaluation

Deoxyribonucleic acid (DNA) was extracted from whole blood PMBC for an index patient (subject II-4) and her mother (subject I-2). Using the SureSelect XT Human All Exon V5 + UTR or V6 + UTR kit (Agilent Technologies, USA), DNA was prepared for a generation of whole-exome sequencing (WES) libraries. Barcoded libraries were sequenced on a NextSeq 500 platform (Illumina, USA) with an average coverage depth of 100 × . Bioinformatics analysis and subsequent filtering identified rare sequence variants. Candidate variants were prioritized based on gene function and relevance to the studied phenotype. Following the WES results, familial segregation for the *FNIP1* variant was performed by Sanger sequencing.

## Results

### Clinical evaluation

**Fig. 1:**
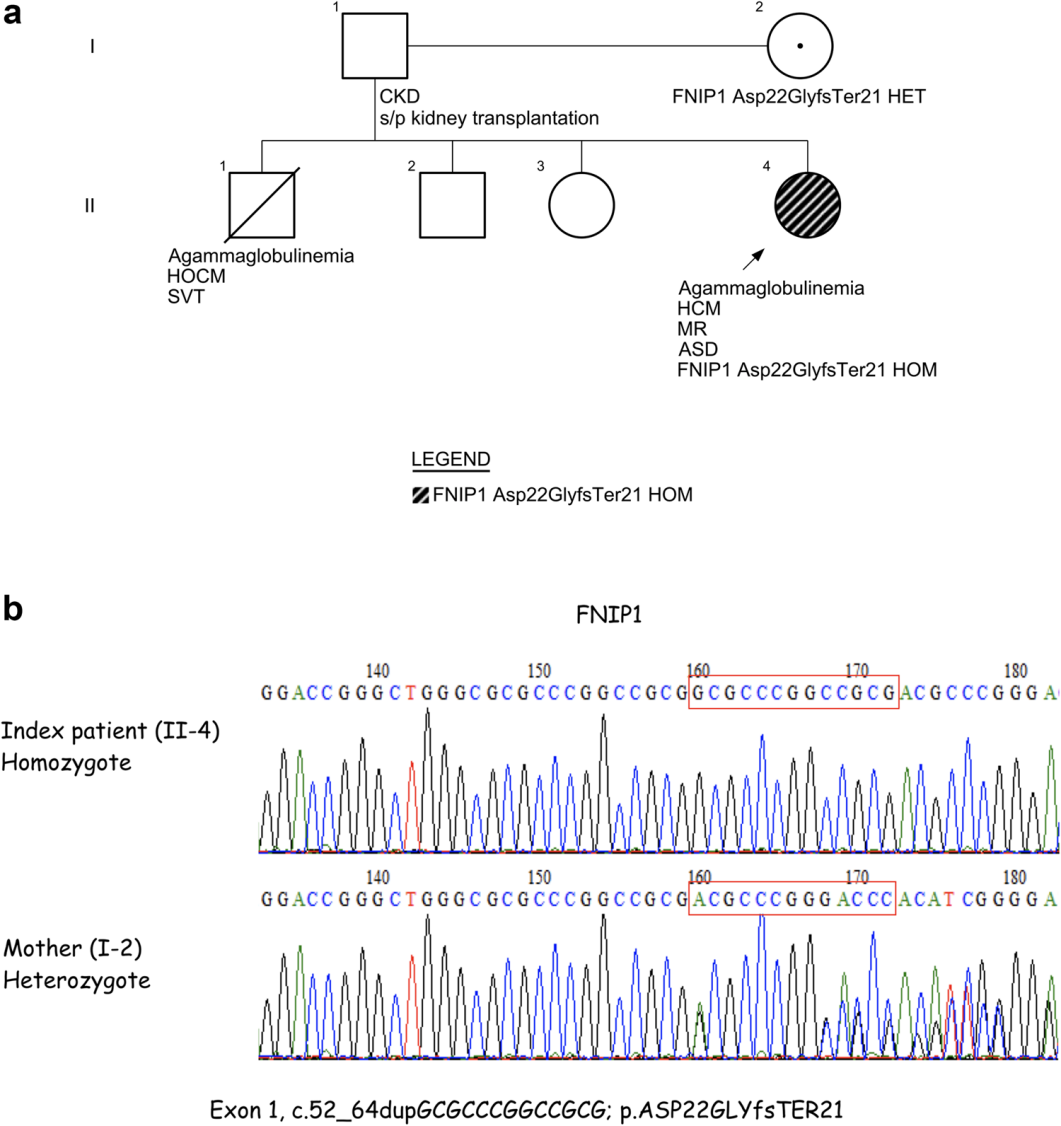
**a** Pedigree of the family studied for the FNIP1 LOF. Abbreviations: Arrow, index patient; ASD, atrial septal defect; CKD, chronic kidney disease; FNIP1, Folliculin interacting protein 1; HCM, hypertrophic cardiomyopathy; HET, heterozygous; HOCM, hypertrophic obstructive cardiomyopathy; HOM, homozygote; MR, mitral regurgitation; SVT, supraventricular tachycardia. **b** Sanger sequencing chromatogram depicting the 13 bp duplication in *FNIP1*

The index patient (subject II-4) was born to non-consanguineous Arab-Muslim parents (Fig. 1a). Shortly after birth, she was diagnosed with atrial-septal defect (ASD), mitral regurgitation (MR), HCM, and left ventricular hypertrophy (LVH) on transthoracic echocardiography, and cardiac computed tomography (CT). At the age of 4 months, she developed recurrent viral and bacterial infections, including recurrent sinopulmonary infections (acute otitis media and pneumonia), cellulitis, and prolonged diarrhea requiring repeated antibiotic treatments and hospitalizations. Accordingly, she developed failure to thrive (FTT). Non-typhi salmonella was isolated from stool cultures. Family history revealed a father (subject I-1) who suffered from chronic kidney disease at an early age which required allogeneic renal transplantation. Further information of his renal disease is unavailable. Her sibling (subject II-1) died in infancy.

At the age of 1 year, the patient was referred to our center for immunological and genetic evaluation. Her physical examination revealed no dysmorphic features. She did not have tonsils or palpable lymph nodes. T cell lymphocyte as well as neutrophil workups were normal (Table 1). Due to complete absence of B lymphocytes, low levels of IgG and undetectable levels of IgA and IgM, treatment with monthly intravenous immunoglobulin (IVIg) infusion was initiated, achieving normal IgG levels and exhibiting marked improvement of infections, as well as a substantial decrease in gastrointestinal symptoms. Due to cardiac involvement, she is also treated with acetylsalicylic acid, captopril, furosemide, and carnitine achieving normal growth and no physical limitations.

The index patient’s sibling (subject II-1) presented shortly after birth with dysmorphic facial features, hypotonia, supra-ventricular tachycardia (SVT) as well as bi-ventricular hypertrophy and hypertrophic obstructive cardiomyopathy (HOCM). He had normal lymphocyte and neutrophil counts, but profound agammaglobulinemia was found (Table 1). Normal brain, abdominal, and renal sonography were documented with no cysts. He died at 2 months old due to cardiogenic shock.

**Table 1:** Clinical, genetic, and immunological workup

	Subject II-4	Subject II-1
Laboratory and immunological evaluation		
**Immunoglobulins (normal values (%)**)		
IgG (540–1340 mg/dl)	**227**	**272**
IgA (30–188 mg/dl)	** < 24**	** < 24**
IgM (56–208 mg/dl)	** < 17**	** < 17**
IgE (0–90 IU/ml)	** < 4.5**	N/A
**Lymphocyte sub-populations** (**normal values (%))**		
ALC (4000–10,500 cells/ mm^3^) (60–85%)	10,207	5460
CD3^+^ (2600–8600 cells/ mm^3^) (60–85%)	8268 (81%)	N/A
CD4^+^ (440–1400 cells/mm^3^) (36–63%)	**4287 (42%)**	N/A
CD8^+^ (160–880 cells/ mm^3^) (15–40%)	**3675 (36%)**	N/A
CD4 + /CD8 + (1.5–3.3)	**1.17**	N/A
CD19^+^ (50–300 cells/ mm^3^) (8–22%)	**0 (0%)**	N/A
CD20^+^ (50–300 cells/ mm^3^) (8–22%)	**0 (0%)**	N/A
CD56^+^ (6–30%)	29%	N/A
CD16 + (6–30%)	29%	N/A
**T-cell functional tests**		
**TCR-VB repertoire**	Polyclonal	N/A
**TREC (> 400 copies)**	10,925	N/A
**Neutrophils evaluation** (**normal values (%))**		
Absolute neutrophil count (1500–8500 cells/mm^3^)	5890	1720
Dihydrorhodamine (DHR) analysis	1. PMA-normal 2. *E. coli*-low	N/A

Abbreviations: bold font, abnormal values; *ALC*, absolute lymphocyte count; *ANC*, absolute neutrophil count; *CD*, cluster of differentiation; *E. coli*, Escherichia coli; *Ig*, immunoglobulin; *N/A*, not available; *PMA*, phorbol myristate acetate; *TCR*, T-cell receptor; *TREC*, T-cell receptor excision circles

### Genetic evaluation

The index patient underwent WES which identified a novel homozygous variant in *FNIP1* located on chromosome 5: 131,796,857, NM_133372.3, c.52_64dupGCGCCCGGCCGCG, p. (Asp22GlyfsTer21) resulting in a frameshift of exon 1/18. According to the ACMG criteria (Richards et al. 2015), the variant is classified as likely pathogenic. The novel *FNIP1* variant is a 13-bp duplication, causing a frameshift and eventually an early stop codon. The pLI score (gnomAD) was 1 (maximal score, indicating pathogenicity). Segregation studies employing Sanger sequencing identified the mother (subject I-2) as a heterozygous carrier for the identified variant (Fig. 1b). Currently, genetic data is unavailable for the father (subject I-1) and the other siblings (subjects II-2 and II-3).

## Discussion

Great importance lies in the identification and characterization of novel IEI, promoting our understanding of the normal cellular biology and physiology (Kwon et al. 2023; Spivak et al. 2024). Establishing a genetic diagnosis offers numerous benefits for both patients and their families. It facilitates a better understanding of clinical manifestations, enables the development of personalized molecular and even genomic-based therapeutic strategies, and, importantly, provides patients and families with appropriate genetic counseling (Rae et al. 2018; Seleman et al. 2017; Simon et al. 2020; Somekh et al. 2024).

In this study, we describe a patient found to harbor a novel homozygous variant in *FNIP1*, located in exon 1, resulting in B-cell lymphopenia, agammaglobulinemia, recurrent infections, and cardiomyopathy. We could only assume that her deceased sibling (subject II-1) who exhibited a similar phenotype including profound agammaglobulinemia and severe cardiac involvement, eventually succumbing of cardiogenic shock, very likely harbored an identical genetic variant in *FNIP1*. A healthy mother (subject I-2) was found to harbor a heterozygous mutation. The genetic status of the father (subject I-1) was unfortunately unavailable (Fig. 1a).

The findings of the index patient and her deceased sibling are consistent with previous reports in the literature. Saettini et al. (2021) described 3 patients harboring homozygous *FNIP1* genetic variants. Patient 1 harbored a homozygous nonsense *FNIP1* variant (NM_133372.2; c.868C > T), patient 2, a homozygous splice site variant (c.3306 + 1G > A), and patient 3, a large deletion in exons 9 to 18 and a paternally inherited single-nucleotide variant (c.3218delT; p. Leu1073Wfs*32) causing agammaglobulinemia, neutropenia, recurrent infections along with HCM, renal cysts, and neurological disabilities. Niehues et al. (2020) described 3 patients with homozygous *FNIP1* variants causing hypogammaglobulinemia, recurrent sinopulmonary and gastrointestinal infections, cardiac involvement with HCM, Wolff-Parkinson-White (WPW) syndrome, and metabolic myopathy in a single patient. Moreno‑Corona et al. (2023) described an additional FNIP1-deficient patient presenting with hypogammaglobulinemia, recurrent infections, enteropathy, HCM, and WPW. Most patients were diagnosed in childhood, whereas others were diagnosed at an older age. Cardiac involvement was the most prominent feature in some patients, while others exhibited more significant neurological manifestations. Additionally, some patients had more pronounced immunodeficiency, recurrent infections, and enteropathy. The prognosis for FNIP1 LOF patients remains uncertain, and extended follow-up is necessary for a clearer understanding (Moreno-Corona et al. 2023; Niehues et al. 2020; Saettini et al. 2021).

The index patient in our study (subject II-4) exhibited several clinical and laboratory features consistent with previously reported cases of FNIP1 LOF variants, including agammaglobulinemia, recurrent infections, chronic diarrhea, and cardiomyopathy. Neutrophil counts and function, assessed by oxidative burst with dihydrorhodamine (DHR) analysis, were normal in response to phorbol-myristate-acetate (PMA) but low in response to *E. coli* stimulation, in contrast to previous reports of neutropenia in other *FNIP1* variants (Saettini et al. 2021), and no renal or liver cysts were found on sonography. Her sibling (subject II-1) presented with similar clinical manifestations, alongside additional features such as dysmorphic facial traits and hypotonia. Based on these findings, we believe that the homozygous novel variant in *FNIP1* is the likely cause of the observed clinical phenotype.

## Conclusions

This study demonstrates the significance of providing a timely genetic diagnosis of patients with IEI, for genetic counseling and appropriate treatment. FNIP1 LOF should be considered in patients presenting during infancy with cardiac manifestations along with agammaglobulinemia (and B-cell lymphopenia).

## Data Availability

Data is available in a repository and can be accessed via a DOI link.
